# Tricky Isomers—The Evolution of Analytical Strategies to Characterize Plasmalogens and Plasmanyl Ether Lipids

**DOI:** 10.3389/fcell.2022.864716

**Published:** 2022-04-27

**Authors:** Jakob Koch, Katrin Watschinger, Ernst R. Werner, Markus A. Keller

**Affiliations:** ^1^ Institute of Human Genetics, Medical University of Innsbruck, Innsbruck, Austria; ^2^ Institute of Biological Chemistry, Biocenter, Medical University of Innsbruck, Innsbruck, Austria

**Keywords:** ether lipid biosynthesis, mass spectrometry, phospholipid analytics, PEDS1, plasmalogen physiology, plasmenyl and plasmanyl isomers

## Abstract

Typically, glycerophospholipids are represented with two esterified fatty acids. However, by up to 20%, a significant proportion of this lipid class carries an ether-linked fatty alcohol side chain at the *sn*-1 position, generally referred to as ether lipids, which shape their specific physicochemical properties. Among those, plasmalogens represent a distinct subgroup characterized by an *sn*-1 vinyl-ether double bond. The total loss of ether lipids in severe peroxisomal defects such as rhizomelic chondrodysplasia punctata indicates their crucial contribution to diverse cellular functions. An aberrant ether lipid metabolism has also been reported in multifactorial conditions including Alzheimer’s disease. Understanding the underlying pathological implications is hampered by the still unclear exact functional spectrum of ether lipids, especially in regard to the differentiation between the individual contributions of plasmalogens (plasmenyl lipids) and their non-vinyl-ether lipid (plasmanyl) counterparts. A primary reason for this is that exact identification and quantification of plasmalogens and other ether lipids poses a challenging and usually labor-intensive task. Diverse analytical methods for the detection of plasmalogens have been developed. Liquid chromatography–tandem mass spectrometry is increasingly used to resolve complex lipid mixtures, and with optimized parameters and specialized fragmentation strategies, discrimination between ethers and plasmalogens is feasible. In this review, we recapitulate historic and current methodologies for the recognition and quantification of these important lipids and will discuss developments in this field that can contribute to the characterization of plasmalogens in high structural detail.

## 1 Introduction to Plasmalogens and Other Ether Lipids

**FIGURE 1:**
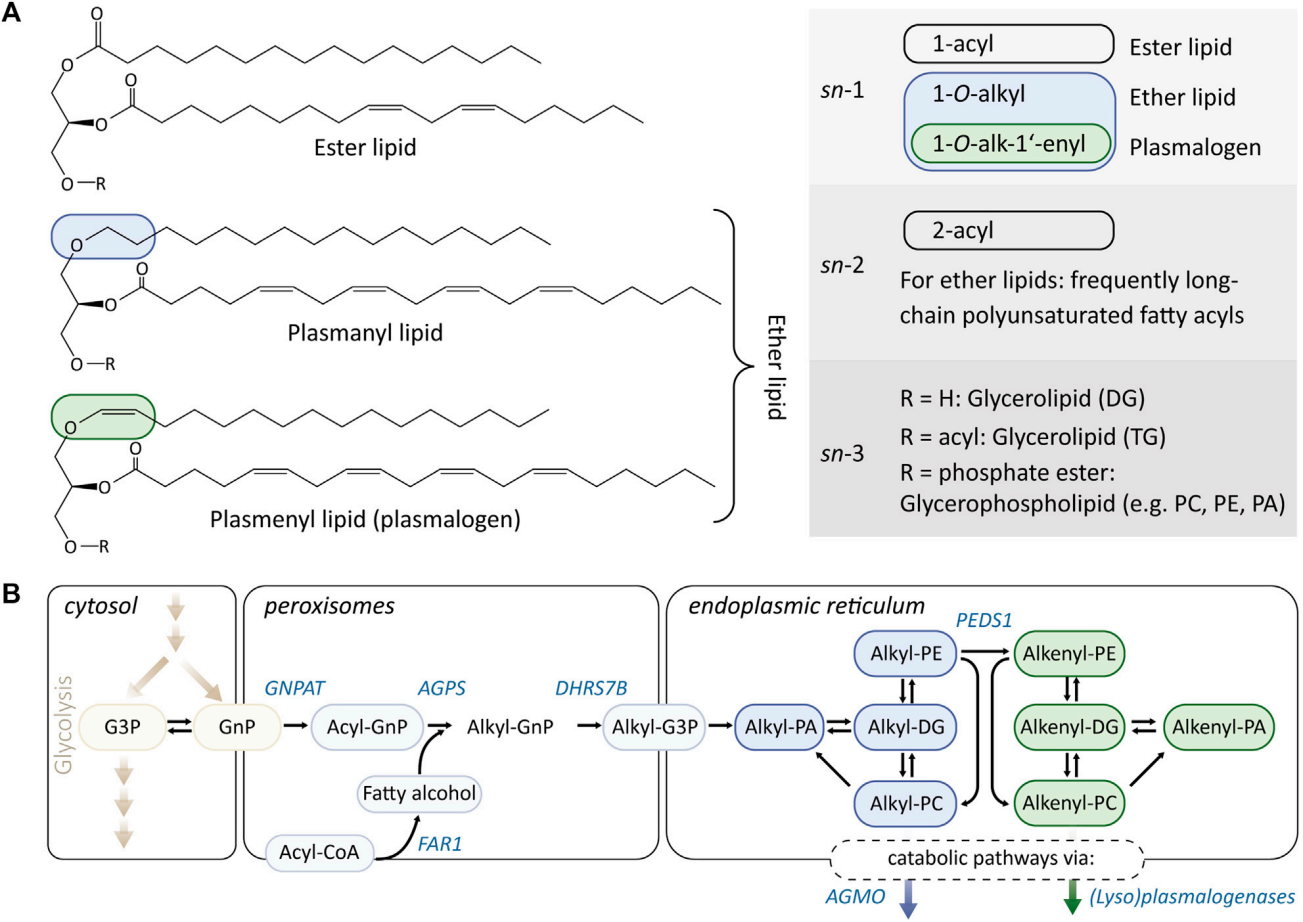
**(A)** Overview of the chemical structures and unique features of diacyl (ester) lipids, and plasmanyl and plasmenyl lipids. Ether lipids comprise lipids that harbor both alkyl (1-*O*-alkyl, plasmanyl, indicated in blue) and alkenyl (1-*O*-alk-1′-enyl, plasmenyl, indicated in green) residues, while the latter is also referred to as plasmalogens. The alkyl/alkenyl residues of mammalian ether lipids are predominantly localized at the *sn*-1 position, while the *sn*-2 position is frequently substituted with polyunsaturated fatty acyls. Ether lipids belong for the most part to the lipid classes phosphatidylethanolamines (PEs), phosphatidylcholines (PCs), and 1-*O*-alkyl-2-acylglycerols (alkyl-DG). **(B)** Ether lipid metabolism. The initial steps of ether lipid biosynthesis take place in the peroxisomes and are based on substrates derived from glycolysis and fatty acid metabolism. The rate-determining step is the provision of fatty alcohols, which are formed from acyl-CoA by fatty acid reductase 1 (FAR1). Remodeling/interconversion pathways of ether lipids are partially shared with their ester lipid analogs. The enzyme plasmanylethanolamine desaturase (PEDS1) is responsible for converting plasmanyl lipids (blue) into their plasmenyl counterparts (green) and accepts PE as substrates. The catabolism of ether lipids proceeds from their lyso-forms and is catalyzed by alkylglycerol monooxygenase (AGMO; in the case of plasmanyl lipids) and (lyso)plasmalogenases (in the case of plasmenyl lipids).

Many complex lipids are made up of simple lipid building blocks such as fatty acids. In the case of glycerophospholipids, these fatty acids are derivatized to a glycerol backbone as fatty acyl esters (Figure 1A). However, besides this esterification, other linkage types also exist that lead to structurally distinct subclasses of glycerophospholipids with divergent physicochemical properties and cellular functions. The so-called ether lipids carry ether-linked fatty alcohols instead of fatty acyls (Figure 1A). A well-known subgroup of ether lipids are plasmalogens, which are characterized by a vinyl ether double bond (alkenyl) instead of an ether bond (alkyl). Consequently, every plasmalogen can be regarded as an ether lipid, while not every ether lipid is a plasmalogen.

### 1.1 Occurrence and Molecular Composition of Plasmanyl and Plasmenyl Lipids

In mammals, the ether bond of plasmalogens and other ether lipids is predominantly located at the *sn*-1 position of the glycerol backbone (Marinetti and Erbland, 1957; Rapport et al., 1957; Debuch, 1958), typically substituted with a saturated or mono-unsaturated alkyl/alkenyl residue, such as palmitoyl, stearyl, and oleyl alcohols (Debuch, 1958). In contrast, the fatty acyls of ether lipids, which are generally derivatized to the *sn*-2 position, are frequently long-chained (≥20) and polyunsaturated (Arthur et al., 1985). The exact fatty acyl composition strongly depends not only on the respective organisms (Vítová et al., 2021) but also follows a pronounced tissue-specificity (Koch et al., 2020). The main substituents at the *sn*-3 position of the glycerol backbone are ethanolamine and choline, generating ether-linked phosphatidylethanolamines (PEs) and phosphatidylcholines (PCs), respectively. However, ether lipid analogs to the neutral di- and triacylglycerides (DG and TG, respectively) exist, which have already been known for decades (Schmid et al., 1967; Snyder and Wood, 1968; Lin et al., 1977) and have recently been getting more and more attention (Schievano et al., 2013; Draijer et al., 2020; Meletis, 2020; Wang et al., 2020; Beyene et al., 2021). Additionally, the presence of small proportions of ether lipid species has also been described for other lipid classes (Ivanova et al., 2010).

Plasmalogens and ether lipids are abundant in animals across invertebrate and vertebrate species (Goldfine, 2010), where they can account for up to 20% of the phospholipid mass, depending on the respective tissues (Nagan and Zoeller, 2001; Braverman and Moser, 2012). Furthermore, plasmalogens are also present in many anaerobic bacteria (Řezanka et al., 2012) and archaea (Jain et al., 2014). However, these lipids typically do not occur in aerobic bacteria (Kamio et al., 1969), fungi (Horrocks and Sharma, 1982), and possibly plants (Felde and Spiteller, 1994).

### 1.2 Biosynthesis and Metabolism of Plasmanyl and Plasmenyl Lipids

As shown in Figure 1B, ether lipids are initially synthesized in peroxisomes (Wanders and Brites, 2010), where the glycolysis intermediate glycerone-phosphate [GnP; previously called dihydroxyacetone phosphate (DHAP)] is first acylated by glycerone-phosphate *O*-acyltransferase (GNPAT). This produces acyl-GnP (which is a precursor for both diacylglycerols and ether lipids) and is followed by replacement of the acyl group for a fatty alcohol by alkylglycerone-phosphate synthase (AGPS) (Nagan and Zoeller, 2001). The so-formed alkyl-GnP is then reduced by an alkylglycerone-phosphate reductase activity (encoded by DHRS7B; also acting on acylglycerone-phosphate) to 1-alkyl-glycero-*sn*-3-phosphate (alkyl-G3P) and exported from the peroxisomes. The availability of fatty alcohols is thought to be the rate-limiting factor in ether lipid and plasmalogen biosynthesis and is controlled by fatty acid reductase 1 and 2 (FAR1/2) (Cheng and Russell, 2004; Honsho et al., 2010; Ferdinandusse et al., 2021). A series of lipid metabolic enzymes that catalyze reactions at the *sn*-2 and *sn*-3 positions of glycerophospholipids are thought to also accept their ether lipid analogs as substrates and are responsible for generating the main ether lipid classes 1-*O*-alkyl-2-acyl-glycerol, alkyl-PE, and alkyl-PC. The formation of plasmalogens is catalyzed by the enzyme plasmanylethanolamine desaturase (PEDS1), which is capable of introducing the vinyl ether double bond at the Δ1 position of an alkyl-PE. Despite its central position in plasmalogen biosynthesis, the gene coding for PEDS1 has only recently been identified (Gallego-García et al., 2019; Werner et al., 2020; Wainberg et al., 2021). In contrast to the molecular oxygen requiring PEDS1, the anaerobic biosynthetic pathway of plasmalogens that has been characterized recently operates in an oxygen independent manner (Jackson et al., 2021). This is in line with the hypothesis that the capability of species to synthesize plasmalogens was once lost during evolution and only later reemerged in eukaryotes (Goldfine, 2010). Once formed, plasmalogens follow a different catabolism regime from other ether lipids. While the alkyl bond of ether lipids is cleaved by the tetrahydrobiopterin-dependent enzyme alkylglycerol monooxygenase (AGMO) (Watschinger et al., 2010), the removal of an alkenyl residue requires specialized (lyso)plasmalogenases (Warner and Lands, 1961; Jenkins et al., 2018). However, the plasmanyl and plasmenyl catabolic pathways both form fatty aldehydes that are toxic to cells if not readily oxidized by the enzyme fatty aldehyde dehydrogenase (FALDH) (Keller et al., 2014; Weustenfeld et al., 2019). Genetic impairment of FALDH function leads to the inherited metabolic disease Sjögren–Larsson Syndrome (SLS) (Weustenfeld et al., 2019), in which fatty aldehydes are interconverted into fatty alcohols, instead of fatty acids (Rizzo and Craft, 2000; Keller et al., 2012). This represents a FAR1-independent source of fatty alcohols that can induce the biosynthesis of ether lipids, which accumulate, for example, in the brain of SLS patients (Staps et al., 2020). Further significant crosstalk has been reported between ether lipid metabolism and other lipid classes such as cholesterol and sphingolipids, indicating that the metabolic routes shown in Figure 1B are additionally deeply rooted in the regulation of cellular phospholipid homeostasis (Braverman and Moser, 2012; Dean and Lodhi, 2018; Harayama and Riezman, 2018).

### 1.3 Physiological Roles of Plasmanyl and Plasmenyl Lipids

The full functional spectrum of plasmalogens and other ether lipids is still far from being comprehensively elucidated. However, it is clear that they are structural components of cellular membranes across a broad range of different tissues (Braverman and Moser, 2012) because ether lipids are found in the plasma membrane and in different subcellular compartments (Sun, 1973; Kuerschner et al., 2012). Due to their frequently polyunsaturated fatty acid (PUFA)-rich *sn*-2 side chains, they are considered to be an important reservoir for lipid second messenger precursors (Nagan and Zoeller, 2001). In previous research studies, particular attention has been paid to the specific properties of the vinyl ether bond of plasmalogens, which was demonstrated to be much more susceptible to oxidative cleavage than analog ester lipids (Broniec et al., 2011). Because of that, and due to their high PUFA content, plasmalogens are considered to be efficient membrane-localized antioxidants (Brites et al., 2004; Engelmann, 2004). More recently (however due to the same physicochemical properties), ether lipids have been implicated in the promotion of and robustness against ferroptosis (Zou et al., 2020). Some individual ether lipid species have been found to encompass highly specific functions. An important representative is certainly the platelet-activating factor (PAF), a 1-*O*-alkyl-2-acetyl-*sn*-glycero-3-phosphocholine which acts as a highly potent intracellular signaling molecule, that is involved in the regulation of many cellular processes [discovered in Demopoulos et al. (1979) and reviewed in Snyder (1999) and Lordan et al. (2019)]. Another example is the ether lipid seminolipid - besides sulfatide the only other major sulfoglycolipid - which is mainly synthesized in primary spermatocytes and essential for spermatogenesis (Goto-Inoue et al., 2009). A third important example are glycosylphosphatidylinositol (GPI) anchors that posttranslationally attach more than 250 different eukaryotic proteins to the surface of membranes (Paulick and Bertozzi, 2008). A neglected aspect in many functional studies on ether lipids is the clear differentiation between the potentially different biological functional spectrum of plasmanyl and plasmenyl lipids (Jiménez-Rojo and Riezman, 2019).

## 2 The Analytical Challenge

Novel breakthroughs and findings in the field of plasmalogen and ether lipid research are strongly linked to the available analytical possibilities. An overview of respective developments throughout the last century is provided in the Section 3. In the past until today, reliable differentiation between plasmanyl and plasmenyl lipids represents a major analytical challenge that determines the pace of scientific progress. An important analytical principle that can be exploited for the quantification of ether lipids is that their alkyl and alkenyl residues are nonsaponifiable. Furthermore, the vinyl ether bond of plasmalogens—but not the ether bond of all other ether lipids—can be cleaved under acidic conditions, thereby yielding the respective fatty aldehydes (Werner et al., 2018). Subsequent derivatization of the released aldehydes allows for the quantification of plasmalogen levels; however, the disadvantage is that the presence of free fatty aldehydes, produced from other sources, can significantly distort the validity of the results. With measurement methods based on this principle, the concentrations of plasmanyl ether lipids remain completely obscure.

In recent years, the use of liquid chromatography–tandem mass spectrometry (LC-MS/MS) has become increasingly popular, also for ether lipid analytics. Despite the great possibilities this technology offers for characterizing complex lipid mixtures, ether lipids still remain a challenging class of analytes, as will be discussed in Section 4. Specifically, it is not possible to univocally differentiate between monounsaturated plasmanyl and saturated plasmenyl residues based on exact mass-to-charge ratios and fragment spectra alone (Koch et al., 2020). This challenge can be overcome by employing specialized instrumentation and techniques (Section 3, 4). Another possibility is to exploit the differential chromatographic behavior of isobaric plasmanyl and plasmenyl lipids, which can, however, not always readily be integrated due to the lack of sufficiently complete sets of commercially available standards (Koch et al., 2020). One property that eases the analysis of molecular ether lipid species is that their structural variability is less diverse than that of other phospholipid classes (Keller, 2021) because at the *sn*-1 position only a limited set of relevant fatty alcohols is found (Cheng and Russell, 2004), while the *sn*-2 position is often occupied by polyunsaturated fatty acids (Arthur et al., 1985; Koch et al., 2020). A further aspect that represents a major challenge in the research of plasmalogens and other ether lipids is that our knowledge about the respective metabolic pathways for a long time (and partially still) showed substantial gaps because the genes of several important enzymes were not known. This rendered the establishment of suitable model systems very difficult.

## 3 History of Ether Lipid Research and the Progress in Ether Lipid Analytics

### 3.1 Discovery and Characterization of Ether Lipids

**FIGURE 2:**
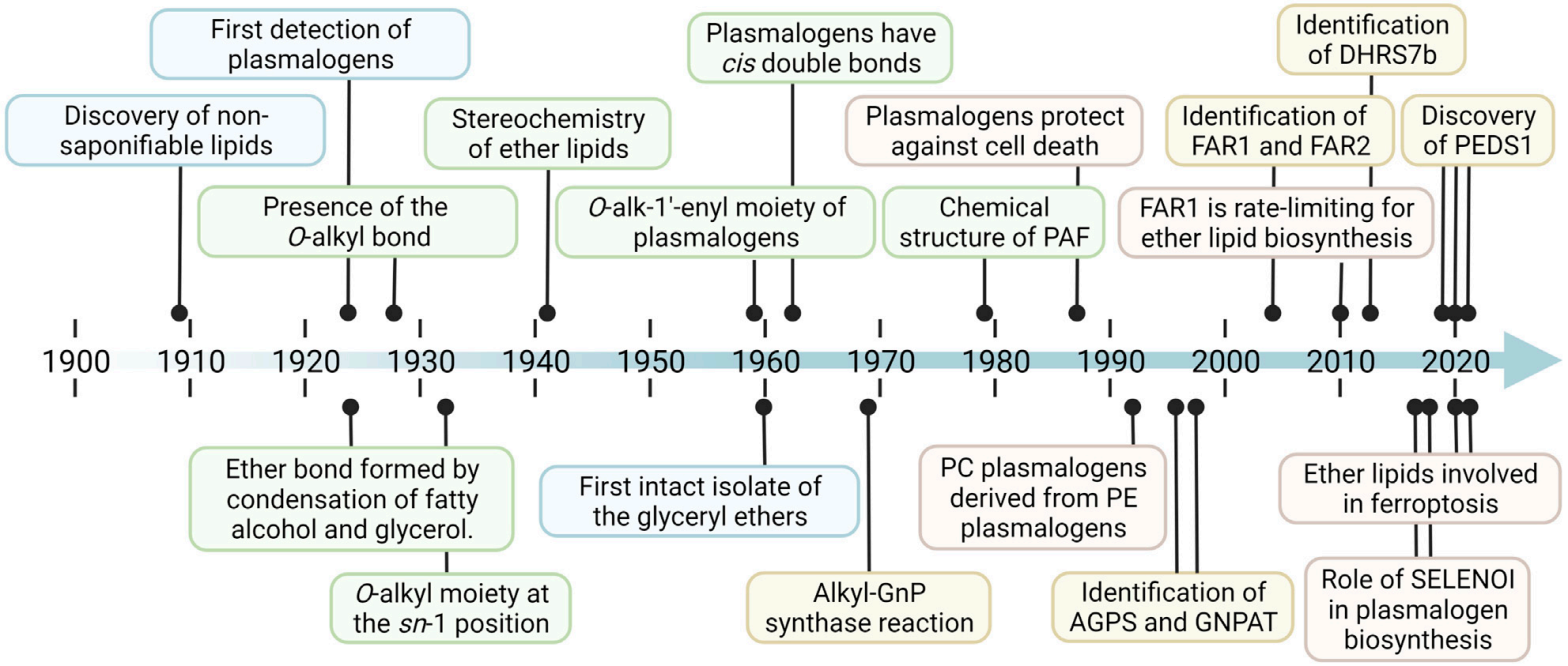
Timeline of selected noteworthy discoveries and milestones in plasmalogen and ether lipid research. In the more than a hundred years since the first evidence for the existence of ether lipids was found, there have been groundbreaking results on a wide variety of conceptual levels. These include their 1) discovery (blue), 2) structural characterization (green), 3) enzymology and metabolism (yellow), and 4) function and physiological roles (red). Corresponding references and additional pioneering findings are listed in Table 1.

The first evidence for the existence of ether lipids was published in 1909, in which the presence of a nonsaponifiable lipid fraction in isolates from starfish was established (Dorée, 1909). In the more than 100 years that followed, a whole series of breakthroughs led to our current understanding of ether lipids and plasmalogens, a selection of which is represented in Figure 2 (for references, Table 1). Fred Snyder, without a doubt one of the most central figures in the advancement of the field, provided a detailed personal and historical perspective on many of these developments up to the turn of the millennium (Snyder, 1999). Until the 1970s, a majority of the research activities relating to plasmalogens focused on the elucidation of their fundamental structure and composition. Later on, important further discoveries were made, such as solving the exact chemical structure of the platelet-activating factor (PAF) in 1979 (Benveniste et al., 1979; Blank et al., 1979; Demopoulos et al., 1979). In parallel, but especially starting from the late 1950s, increasing attention was paid to the natural occurrence of ether lipids and plasmalogens in a wide variety of species, tissues, and other diverse biological sources (Carter et al., 1958; Hanahan and Watts, 1961; Gross, 1985). The growing understanding of plasmalogens and ether lipids triggered an era of research on their metabolism, biochemistry, and biological functions. The first signs of this change in research focus appeared in the 1960s (Kiyasu and Kennedy, 1960; Wykle and Snyder, 1969; Zoeller et al., 1988; Blank et al., 1993), a strong intensification of efforts was especially noticeable from the late 1980s onward. However, many research questions still remain unanswered, particularly regarding the enzymology of the metabolic network related to ether lipids and plasmalogens. Surprisingly, for a long time, it was not possible to identify the genes for many of the enzymes involved in their metabolism, even after the human genome had been deciphered. For example, only recently the genes coding for important functions such as the catabolism of plasmalogens (Wu et al., 2011), the *sn*-1 cleavage of plasmanyl lipids (Watschinger et al., 2010), and the core enzyme responsible for plasmalogen biosynthesis were identified (Gallego-García et al., 2019; Werner et al., 2020; Wainberg et al., 2021). In ether lipid metabolism other orphan enzymes might be present, but it is unclear so far whether the respective ester metabolizing enzymes that are already known also accept the ether analogs as substrates.

**TABLE 1:** Milestones in plasmalogen research [expanded on the basis of Snyder (1999)].

Year	Milestone	Citation
1909	Nonsaponifiable lipid isolates from starfish	Dorée (1909)
1924	Condensation of a long-chain fatty alcohol and glycerol forms an ether bond	Toyama (1924)
1924	First detection of plasmalogens	Feulgen and Voit (1924)
1928	Presence of an *O*-alkyl bond	Heilbron and Owens (1928)
1933	*O*-Alkyl moiety located at the *sn*-1 position	Davies et al. (1933)
1941	Stereochemistry of ether lipids	Baer and Fischer (1941)
1957	*O*-Alk-1′-enyl moiety of plasmalogens	Rapport et al. (1957); Debuch (1958)
1958	Presence of PE ether lipids in egg yolk	Carter et al. (1958)
1960	First intact isolate of the glyceryl ethers	Mangold and Malins (1960)
1960	Choline and ethanolamine phosphotransferases catalyze the transfer of CDP-choline and CDP-ethanolamine to *O*-alk-1′-enyl-acylglycerols	Kiyasu and Kennedy (1960)
1961	Isolation of ether lipids from bovine erythrocytes	Hanahan and Watts (1961)
1962	*cis*-nature of plasmalogen double bond	Norton et al. (1962); Warner and Lands (1963)
1963	Double bond position in the *O*-alkyl chain of selachyl alcohol at carbons 9 and 10	Hanahan et al. (1963)
1969	Cell-free synthesis of *O*-alkyl bond	Snyder et al. (1969)
1969	Alkyl-DHAP synthase forms ether lipids from glycerone-phosphate and a fatty alcohol	Hajra (1969); Wykle and Snyder (1969)
1979	Discovery of the chemical structure of platelet-activating factor (PAF)	Benveniste et al. (1979); Blank et al. (1979); Demopoulos et al. (1979)
1985	Plasmalogens are the major phospholipid constituent of the cardiac sarcoplasmic reticulum	Gross (1985)
1988	Plasmalogen bond protects against cell death	Zoeller et al. (1988)
1993	Choline plasmalogens are mainly derived from the ethanolamine plasmalogens	Blank et al. (1993)
1997	Identification of *AGPS*	de Vet et al. (1997)
1997/1998	Identification of *GNPAT*	Thai et al. (1997); Ofman et al. (1998)
2004	Identification of *FAR1* and *FAR2*	Cheng and Russell (2004)
2010	FAR1 is the rate-limiting enzyme in ether lipid biosynthesis	Honsho et al. (2010)
2012	Identification of *DHRS7b*	Lodhi et al. (2012)
2017	Identification of *EPT1* (*SELENOI*)	Ahmed et al. (2017)
2018	Role of *SELENOI* for plasmalogen biosynthesis	Horibata et al. (2018)
2019/2021	Identification of *PEDS1*	Gallego-García et al. (2019); Werner et al. (2020); Wainberg et al. (2021)
2020/2021	Role of ether lipids in ferroptosis	Zou et al. (2020); Cui et al. (2021)
2021	Plasmalogen synthase in anaerobic bacteria	Jackson et al. (2021)

### 3.2 The Role of Novel Analytical Techniques

A major driving force behind the scientific progress in ether lipid and plasmalogen research was (and is) the different accessible analytical technologies. Importantly, the analytical tools available at the respective time also had a decisive influence on the trajectory of the research activities (and *vice versa*). Several methods for quantifying plasmalogen concentrations rely on the cleavage of the vinyl ether double bond in the presence of an acid, and the detection of the liberated aldehyde, frequently as an acetal or hydrazone derivative (Figure 2 and Table 1). While the special reactivity of plasmalogens increases their specificity, information on the remaining structure of the molecule, that is, the residues at *sn-*2 and *sn-*3 of the glycerolipids, is lost. In addition, proper controls need to be included to subtract the amounts of free aldehydes present in the sample before acidic liberation of the aldehyde at *sn-*1. This can be done by using hydrochloric acid to cleave the vinyl ether double bond and running an additional reaction with acetic acid instead of hydrochloric acid in parallel, which leaves the vinyl double bond intact and therefore represents the free aldehyde content only (Werner et al., 2018). However, in the selected mouse tissues investigated so far, the amount of free aldehydes was always below 1% as compared to the amount of plasmalogens (Werner et al., 2018). An additional drawback of these methods is that they fail to quantify ether lipids without the vinyl ether double bond, that is, plasmanyl lipids.

Already the very first detection of plasmalogens in 1924 relied on such a reaction, the formation of an adduct of aldehydes liberated from plasmalogens with fuchsin in sulfuric acid (Feulgen and Voit, 1924). A related procedure allowed high-throughput screening for bacterial colonies lacking plasmalogen formation in the search for genes responsible for plasmalogen formation in anaerobic bacteria (Jackson et al., 2021). The formation of dimethyl acetals by cleavage in acidic methanol (Gray, 1969) is still frequently used to quantify plasmalogens by gas chromatography–mass spectrometry (GC-MS) and liquid chromatography–mass spectrometry (LC-MS) methods (Ingrand et al., 2000; Moraitou et al., 2008; Brites et al., 2009; Bueno et al., 2012; Gallego-García et al., 2019). Other methods used 2, 4-dinitrophenyl hydrazine and thin layer chromatography (Rhee et al., 1967), or staining of thin-layer chromatograms with 4-amino-5-hydrazino-1,2,4-triazole-3-thiol sprays (Rahn and Schlenk, 1973). Derivatization with (pentafluorobenzyl)hydroxylamine hydrochloride allowed analysis using GC-MS (Ingrand et al., 2000). We developed a method to measure plasmalogens using dansylhydrazine as a derivatization agent and reversed-phase HPLC with fluorescence detection for quantification (Werner et al., 2018). This method was essential for independent measurement of total plasmalogen in mice deficient in PEDS1 (Werner et al., 2020), enabling the validation of LC-MS methods for the unequivocal discrimination between plasmanyl and plasmenyl lipids (Koch et al., 2020; Werner et al., 2020).

Besides the aforementioned thin layer chromatographic techniques with varying detection reagents and principles for the semi-quantitative analysis of ether lipids and plasmalogens (Schmid et al., 1975; Shantha and Napolitano, 1998), and other chromatographic approaches (Christie and Han, 2012), a diversity of analytical approaches has been developed (Messias et al., 2018). A series of assays based on radiolabeled substrates have been established, often to study the respective metabolic pathway structures and enzymology in a targeted manner (Paltauf, 1972; Wykle et al., 1972; Blank and Snyder, 1992). Furthermore, also ^1^H, ^13^C, and especially ^31^P nuclear magnetic resonance (NMR) approaches have been employed to quantify PC and PE plasmalogens on the basis of the characteristic chemical shifts that vinyl ether double bonds cause (Meneses and Glonek, 1988; Sacchi et al., 1995), a technology that provides new scientific insights (Kimura et al., 2018; Bozelli et al., 2020). A major disadvantage of many of these methods, is that only little or no information about the respective molecular species, especially the chemical structure of their side chains, is extractable. In addition, the methods used often either do not allow a clear distinction between plasmalogens and other ether lipids or can solely quantify plasmalogens. Overcoming this often requires a laborious combination of different methods, such as the saponification of previously extracted and pre-separated ether lipids followed by GC-MS analysis of the fatty acids released (Maulik et al., 1993). For this reason, the analysis of ether lipids by means of LC-MS/MS is increasingly pursued in light of the rapidly improving instrumental performance in the field of mass spectrometry. LC-MS/MS-based approaches are highly attractive due to the abundance of extractable information, but of course, they come along with their specific challenges and problems, especially in regard to the reliable identification of plasmanyl and plasmenyl species, which are summarized in **Section 4**.

### 3.3 Implications for the Pathophysiological Knowledge About Ether Lipids

These and other conceptual and technical advances have of course had a major impact on our understanding of the role of plasmanyl and plasmenyl lipids in health and disease. A detailed summary would go far beyond the scope of this work and is already part of excellent reviews such as Braverman and Moser (2012) and Dean and Lodhi (2018). The functional involvement of ether lipids has been discussed in many pathologies. These include inherited peroxisomal disorders, often caused by mutations in one of the 14 different *peroxin* (*PEX*) genes (Berger et al., 2016) that lead to different manifestations of the Zellweger syndrome, as well as in the case of *PEX7* to rhizomelic chondrodysplasia punctata (RCDP) (Waterham and Ebberink, 2012). Additionally, reduced plasmalogen levels have been associated with neurodegenerative diseases such as Alzheimer’s disease (Dorninger et al., 2020) and are discussed as a potential treatment target (Fujino et al., 2017). Furthermore, plasmalogens were recognized for their protective role against oxidative stress (Zoeller et al., 1988) and have been shown to inhibit apoptosis (Yamashita et al., 2015). However, this behavior has been reported to be cell-type specific, as plasmalogens play an important role in the apoptotic behavior of mouse neuroblastoma-derived cells but not in astrocyte-derived cells (Hossain et al., 2013).

## 4 Mass Spectrometry-Based Ether Lipid Analysis

Historically, characterizing the composition of alkyl and alkenyl lipids with reliable molecular subspecies resolution has been a highly laborious and tedious task, as detailed in the previous section. With the advent of omics technologies and related bioinformatics capabilities, each of which is entangled with the developments in mass spectrometry (MS) instrumentation, the discriminative power and data quality were propelled forward. This is also related to the generation of large, information-rich data sets, where reliable data analysis strategies play a crucial role in systematically deciphering complex lipid compositions. There are a broad range of possibilities and different setups, where MS is used as the core detection principle for the quantification of plasmalogens and other ether lipids. Vítová et al. have recently provided a comprehensive overview on plasmalogen analysis methods and how they are used to study these lipids in various species (Vítová et al., 2021). There are both targeted and untargeted mass spectrometric approaches, and both have their specific advantages and disadvantages in terms of reliability, reproducibility, and information content. These are based on either direct infusion (shotgun) lipidomics (Han and Gross, 2005; Surma et al., 2021) or mass spectrometric detection after different types of pre-separation, including gas or liquid chromatography (Mawatari et al., 2007; Fauland et al., 2011; Lísa et al., 2017), capillary electrophoresis (Zhang et al., 2017; Ly et al., 2021), ion mobility separation (Vasilopoulou et al., 2020; Kirkwood et al., 2022), and supercritical fluid chromatography (Lísa et al., 2017; Schoeny et al., 2020). In this section, we will focus on how to tackle the analytical challenge to discriminate between plasmanyl and plasmenyl lipids. This differentiation is of great importance as introduction of a vinyl ether double bond in ether lipids severely alters the physicochemical properties of the molecule (e.g., its oxidizability) and therefore defines the respective functional roles.

### 4.1 Ether Lipid Identification by Mass Spectrometry

In general, a distinction must be made between high-resolution mass spectrometers, where the instrument can determine masses with an accuracy of as low as 0.1 mDa in relation to the exact mass, and low-resolution mass spectrometers that are only accurate to approximately 1 Da (Wallace and McCord, 2020, 254). In the molecular context of lipids, this implies that distinguishing between isobaric lipids such as the pair PE P-36:2 (plasmenyl) and PE 35:3 with respective mass-to-charge ratios (m/z) of 726.5443 and 726.5079 m/z in electrospray ionization (ESI) negative mode is possible only with high-resolution instruments, while the isomeric counterpart PE O-36:3 (plasmanyl, 726.5443 m/z) cannot be readily differentiated from PE P-36:2 even with highest resolution instruments (Keller, 2021). A further challenge is that such non-resolvable mass overlaps also occur between pairs of lipid species with a nominal mass difference of 2 m/z, as is the case for plasmenyl and plasmanyl species with identical side-chain substitution; that is, the M+2 isotopologue of a plasmenyl species interferes with the M+0 peak of the corresponding plasmanyl lipid (type-II isotopic effect) (Höring et al., 2021). This effect is particularly important when significantly larger amounts of plasmenyl species are present, which is often the case with PE ether lipids (Koch et al., 2020). In addition, the possible distortion due to mass overlaps scales with increasing numbers of non-most abundant (natural) isotopes contained within a lipid, which are responsible for changing isotopic intensity distributions (type-I isotopic effect) (Han and Gross, 2001). In other words, with increasing numbers of, for example, carbon atoms, the natural isotope prevalence causes changes in the isotopic distribution from (M+0) to (M+1) and higher isotopes, thus increasing the problem of interference due to signal interference. A type-II isotopic effect, if lipids differ by one double bond, can be (at least theoretically) circumvented when reaching very high mass resolutions (R > 200,000 for PE O-36:2/PE P-36:2); however, a correction using suitable deconvolution approaches during data analysis is possible. In contrast, this is not the case for the aforementioned overlap between isomers, which can only be resolved through combination with additional complementary analytical techniques.

### 4.2 Mass Spectrometry-Based Fragmentation and Derivatization Approaches

Several powerful possibilities to discriminate between plasmanyl and plasmenyl lipids arise from the fragmentation capabilities that many mass spectrometers provide. However, pure MS/MS fragment spectra in the negative ESI mode are not sufficient to achieve a clear assignment of plasmalogens and other ether lipids (Koch et al., 2020). A more advanced approach is the generation of unique fragmentation signatures obtained via repeated collision dissociations of lithiated ether lipid precursor ions in positive ESI mode [(M + Li)^+^, (M-H + 2Li)^+^, and (M-2H + 3Li)^+^] (Hsu and Turk, 2008) restricted to mainly PE and PC, while in negative mode distinction for all major glycerophospholipid classes can be achieved by multistage fragmentation (Hsu and Turk, 2007; Hsu et al., 2014). However, those approaches are limited to mass spectrometers with MS^n^ (*n* > 2), and therefore intrinsically limited in their applicability (Hsu and Turk, 2008). In another method, silver ion adducts of phosphatidylethanolamine plasmalogens enabled their detection *via* neutral loss scans in the positive ESI mode. In the presence of Ag+ ions, a characteristic neutral loss of 141 Da is also predominantly observed for plasmenylethanolamines, and quantification is enabled by differential analysis (Kim et al., 2012). A further possible workflow includes the combination of ozone-induced dissociation (OzID) in the MS^1^ dimension with additional collision-induced dissociation (CID) fragment spectra (MS^2^) acquired in direct infusion (shotgun) workflows boosting lipidome coverage via a higher number of duty cycles (Deeley et al., 2009; Marshall et al., 2019). Depending on the combinations of CID and OzID, the *sn* position of each fatty acyl (FA) (CID/OzID), the double bond positions in the *sn*-1 FA (CID/OzID^2^), and a full characterization (all double bond positions and chain lengths of *sn*-1 and *sn*-2 FA) are possible with (CID/OzID)^2^ (Pham et al., 2014).

Also, derivatization strategies, for example, with iodine/methanol derivatized plasmenyl lipids, enable the differentiation between plasmanyl and plasmenyl species, and in combination with ^13^C_1_–*S*, *S*′-dimethylthiobutanoyl-*N*-hydroxysuccinimide ester derivatization of aminophospholipids, this method can also resolve type-I isotopic effects (Fhaner et al., 2013). Quite recently, a new application was published where the acquisition of fragmentation spectra at three different higher collision energy settings allowed an established computational model to correct and deconvolute different isobaric and isomeric features with different structural compositions (Schuhmann et al., 2019). Theoretically, this approach could also be able to distinguish between plasmanyl and plasmenyl lipids, but this was not discussed by the authors. A general discussion of the various derivatization strategies applied for MS-coupled lipidomics can be found in Hu et al. (2019). Furthermore, when primarily focusing on the quantification of plasmalogens, a distinct fragmentation behavior of plasmenyl PE lipids in the positive ESI mode can be exploited for their structure-specific quantification (Tsugawa et al., 2020; Morel et al., 2021).

### 4.3 Exploiting Different Chromatographic Properties of Plasmanyl and Plasmenyl Lipids

In addition, but also as an alternative to more complex MS^n^ methods, the combination of MS with chromatographic separation methods can in principle be used for the discrimination of plasmanyl and plasmenyl lipids. Generally, normal phase chromatography and hydrophilic interaction liquid chromatography (HILIC) separate lipids in a lipid class-dependent manner, while in reversed-phase chromatography a lipid species-specific separation behavior is facilitated. Both principles are widely applied in lipidomic studies as described by Harrieder et al. (2022), while promising methods utilizing supercritical fluid chromatography for separation are under development (Wolrab et al., 2020; Le Faouder et al., 2021) that should enable class-wise separation of plasmanyl and plasmenyl lipid species, which in comparison with strategies refined for DI methods (necessary to correct for type-II isotopic effects) should allow high-throughput lipidomics with plasmalogen resolution on a whole lipidome scale.

A major limiting factor for the systematic characterization of the separation properties of plasmalogens and other ether lipids is the lack of commercially available standards in sufficient numbers and variety. Pairs of plasmanyl/plasmenyl species rarely occur together in the same sample, precluding mutual relative referencing (Koch et al., 2020). However, with the help of a plasmalogen-deficient mouse model (Werner et al., 2020), it was possible to comprehensively describe that reversed-phase gradients allow for distinguishing between plasmanyl and plasmenyl lipids by a characteristic retention time offset (Koch et al., 2020). Furthermore, this retention time behavior is systematic (in addition to the contributions of double bond content and carbon atom number within fatty acyl side chains) and allows for building predictive models for the retention time behavior of plasmanyl and plasmenyl lipids in a lipid class-wise manner (Vaňková et al., 2022). This allows the challenging mass spectrometric problem of deconvoluting isomeric and isobaric ether lipids to be transformed into a much easier solvable chromatographic and data analysis issue. Even type-II isotopic effects can be readily resolved with baseline separation (Lange and Fedorova, 2020; Vaňková et al., 2022). This principle is also implemented in several targeted multiple reaction monitoring (MRM) and selective reaction monitoring (SRM) assays (Benjamin et al., 2013; Lee et al., 2021), which can be expanded and improved with an increasing variety of commercial standards. In contrast, in HILIC-based methods, the respective lipid class-wise elution behavior (Buré et al., 2013) reduces the potential to separate plasmanyl and plasmenyl species, although they elute prior to the diacyl lipids (Otoki et al., 2017). With such a separation method entirely focusing on the goal to distinguish plasmalogens, it is possible to achieve a clear separation also with HILIC; however, simultaneously there is a tradeoff in respect to the applicability of the method to characterize the general lipidome (Morel et al., 2021), which diminishes general feasibility of HILIC for detailed lipidomics (Lange and Fedorova, 2020).

### 4.4 Correctly Reporting the Level of Structural Identification

Different identification and quantification strategies for the analysis of plasmalogens and other ether lipids result from the current set of utilized LC-MS/MS-based approaches. Since, as discussed earlier, a differentiation between plasmanyl and plasmenyl species cannot be automatically assumed, this must be taken into account for both identification and lipid species nomenclature. When following good practice rules (Köfeler et al., 2021), it is clearly important to consider the level at which identifications take place, which in turn should be reflected in the name of the lipids (Liebisch et al., 2013, 2020). Depending on how conscientiously this is implemented, this leads to lipidomic studies in which a clear assignment of plasmalogens and other ether lipids 1) is not regarded at all, 2) is based on educated guesses, 3) is honestly reflecting the level of identification, or 4) is explicitly executed with one of the approaches detailed previously. Many general lipidomic studies that are based on reversed-phase HPLC separation do not (yet) distinguish between plasmanyl and plasmenyl lipids. Thus, it is advisable to fully utilize the existing analytical potential of untargeted LC-MS/MS-based lipidomics approaches, as already in standard workflows the combined information of exact masses, fragmentation behavior, and a well-characterized retention time behavior would be sufficient to correctly assign the otherwise tricky plasmanyl and plasmenyl isomers.

## 5 Future Perspectives

PE, PC, and DG are the main lipid classes for which ether lipid and plasmalogen analogs have been described and are currently studied. However, ether-linked lipid species have also been found in a range of different other lipid classes, including phosphatidylinositol, phosphatidic acid, phosphatidylserine, and phosphatidylthreonines (Ivanova et al., 2010). Although these occur in comparatively small amounts, they must still be taken into account as part of the lipidome. However, since commercially available standards are already limited for the main lipid classes, this problem is even more pronounced for rarer ether lipid variants.

Likewise, much of the current research focus related to plasmalogens and other ether lipids relies on a relatively small subset of model organisms. Nevertheless, it has been shown that ether lipids can be much more complex in other organisms such as archaea (Albers et al., 2000; Pineda De Castro et al., 2016; Vítová et al., 2021). For example, while in mammals it can be assumed by default that the ether bond is located at the *sn*-1 position, this is far from set in stone in other species (Grossi et al., 2015) and can also become relevant in the analysis of plasmalogens in food (Yamashita et al., 2016). This circumstance must be explicitly taken into account in ether lipid and plasmalogen analytics.

Plasmanyl and plasmenyl ether lipids are increasingly being associated with diseases other than specific inherited metabolic diseases involving peroxisomes (Ferreira et al., 2021). In addition to Alzheimer’s (Han et al., 2001; Igarashi et al., 2011; Kling et al., 2020), Down syndrome (Murphy et al., 2000), and Parkinson’s disease (Dragonas et al., 2009), these also include abnormalities in the plasma of colorectal cancer patients (Liu et al., 2020). However, the use of plasmalogens as early diagnostic biomarkers places particularly strict demands on the performance of the analytical approaches used.

From the point of view of ether lipid and plasmalogen analytics, there are a number of important measures that should be taken in light of these developments. 1) Above all, it is important that the research field focuses on truthfully reporting the exact structural level of analysis of ether lipids, which should also be reflected in the respectively used lipid nomenclature (Liebisch et al., 2013, 2020) and thereby render them compatible with unified computational naming approaches like Goslin (Kopczynski et al., 2020). This aspect should not only be implemented “in-house” but also urged for, for example, in reviewing activities. 2) A further step is to utilize the structural information that is already available in the raw data more comprehensively, to differentiate between plasmanyl and plasmenyl lipids whenever applicable. 3) With increasing demand, it would be a welcome development if a greater diversity of commercial ether lipid and plasmalogen standard substances becomes available. In this regard, the recent identification of the gene that encodes for a key enzyme in plasmalogen biosynthesis can be of great help (Gallego-García et al., 2019; Werner et al., 2020; Wainberg et al., 2021). 4) Last but not least, it can be highly rewarding to continue working on the development and combination of new technologies. Within certain limits, a further increase in the mass resolution of new mass spectrometers can still have positive effects (type-II isotopic effects). There is strong potential for improvement in data analysis, for example, in terms of the utilization of already existing information and by means of sophisticated deconvolution methods. A promising approach could be the integration of techniques such as ion mobility, which for lipids produces a separation behavior similar to that of reversed-phase chromatography in lipids.

As the history of plasmalogen analysis shows, the continuous development of analytical possibilities has resulted in ever greater insights into the chemistry, biochemistry, and physiology of ether lipids. Nevertheless, the precise physiological functions of these lipids are only superficially understood. Particularly, this applies to the delimitation of the functional spectrum between lipid species that contain plasmenyl and plasmanyl residues, respectively. The intensification of research activities in this field, which is also reflected by this special issue, conveys a highly optimistic perspective about possible upcoming breakthroughs, to which plasmalogen analytics will most likely have a significant contribution.
